# Retraction: Exploring the impact of climate technology, financial inclusion and renewable energy on ecological footprint: Evidence from top polluted economies

**DOI:** 10.1371/journal.pone.0323472

**Published:** 2025-05-07

**Authors:** The PLOS One Editors

The *PLOS One* Editors retract this article [1] due to concerns about potential manipulation of the publication process. These concerns call into question the validity and provenance of the reported results. We regret that the issues were not identified prior to the article’s publication.

LW did not agree with the retraction. FY, JL, and NB either did not respond directly or could not be reached.
